# Patients' Global Impression of Change (PGIC) Score Compared to Monthly Migraine Days to Evaluate Treatment Persistence With Anti‐CGRP Monoclonal Antibodies

**DOI:** 10.1002/acn3.70053

**Published:** 2025-04-17

**Authors:** Marina Romozzi, Catello Vollono, Paolo Calabresi, Francesco De Cesaris, Luigi Francesco Iannone

**Affiliations:** ^1^ Dipartimento Universitario di Neuroscienze Università Cattolica del Sacro Cuore Rome Italy; ^2^ Neurologia, Dipartimento di Neuroscienze, Organi di Senso e Torace Fondazione Policlinico Universitario Agostino Gemelli IRCCS Rome Italy; ^3^ Headache Center and Clinical Pharmacology AOU Careggi Florence Italy; ^4^ Department of Biomedical, Metabolic and Neural Sciences University of Modena and Reggio Emilia Modena Italy; ^5^ Digital and Predictive Medicine, Pharmacology, Clinical Metabolic Toxicology‐Headache Center and Drug Abuse, Laboratory of Clinical Pharmacology and Pharmacogenomics AOU Policlinico di Modena Modena Italy

**Keywords:** migraine, outcome, PGIC, PROM

## Abstract

This study assessed whether continued treatment with anti‐CGRP monoclonal antibodies (mAbs) is driven more by reductions in monthly migraine days (MMDs) or patients' global impression of change (PGIC), a patient‐reported outcome. Among 169 patients treated with anti‐CGRP mAbs, 21.3% discontinued due to ineffectiveness. PGIC responders (≥ 5) at month 12 were 69.8%, whereas MMD responders (≥ 50% reduction) were 59.2%. Both were significantly associated with discontinuation (PGIC: *χ* ^2^ = 33.474, *φ* = 0.445; MMD: *χ* ^2^ = 29.884, *φ* = 0.421; *p* < 0.0001). PGIC showed a stronger correlation with discontinuation (*r*_pb_ = 0.541) than MMD reduction (*r*_pb_ = 0.470). These findings highlight PGIC as strongly associated with treatment response, supporting the need for PROMs in evaluating migraine treatment effectiveness.

## 1 Introduction

The effectiveness of the monoclonal antibodies (mAbs) against the calcitonin gene‐related peptide (CGRP) or its receptor (anti‐CGRP mAbs) is well established in both episodic and chronic forms and migraine with and without aura [1].

The introduction of migraine‐specific prophylactic treatments has offered several advantages over standard non‐specific preventive therapies, including higher adherence, fewer adverse events, and fewer interactions between drugs [2, 3]. However, inappropriate patient selection or early discontinuation may lead to higher direct and indirect healthcare costs [4]. Therefore, it is crucial to define the treatment response to these therapies.

Different percentages of non‐responders have been reported in randomized controlled trials (RCTs) and real‐world studies, ranging from 5% to 30%, according to different effectiveness criteria [5, 6].

The definitions of “effectiveness” and, therefore, the “response status” to anti‐CGRP mAbs treatment and whether the same cutoff should be applied in RCTs and observational studies are a matter of debate [7]. The most widely used variables in all studies are monthly headache days (MHDs) and monthly migraine days (MMDs), being easily measurable and having a more specific weight (theoretically) compared to other parameters. However, even a 50% MMD reduction can be associated with a residual clinically relevant burden. Conversely, a 30% reduction may hold clinical significance, particularly for patients with chronic migraine, where even a modest reduction of MHD/MMDs can have a meaningful impact [8]. The current definition of effectiveness may not fully capture outcomes observed in clinical practice, and including a range of alternative measures, such as migraine disability assessment (MIDAS), Headache impact test‐6 (HIT‐6) scores, analgesic usage, and patient‐reported outcomes (PROMs), should be consistently considered.

Patient's Global Impression of Change (PGIC) is emerging as one of the more useful PROMs to evaluate treatment response due to its easy applicability in clinical settings (namely, just one simple question with a 1–7 scale) and its comprehensiveness for patients and clinicians [9, 10]. It has also been introduced in the field of migraine for assessing treatment response to anti‐CGRP drugs [10].

Furthermore, PGIC is validated and standardized, allowing reproducibility and consistency [9, 10].

Herein, with an analysis from a prospective, observational study that evaluated the effectiveness and safety of anti‐CGRP mAbs in clinical practice and their pattern of discontinuation [11], we evaluate whether continuing treatment with anti‐CGRP mAbs was driven more by the reduction in MMDs compared to their reported impression of global effectiveness, evaluated by the standardized and validated PGIC score.

## 2 Methods

In this post hoc analysis, we included all consecutive adult outpatients with a diagnosis of migraine according to ICHD‐3 criteria, treated with anti‐CGRP mAbs (subcutaneous anti‐ligand and anti‐receptor) with a potential 12‐month follow‐up, including patients who discontinued for ineffectiveness but not for other reasons (i.e., lost to follow‐up, adverse events) due to the study aim.

The study was reported according to the Strengthening the Reporting of Observational Studies in Epidemiology (STROBE) guidelines and is part of the *Registro Italiano Cefalee* (RICe) study, which was approved by the local Ethics committee (CEAVC Studio RICe, 14591_oss and subsequent amendments 2022‐609; 2023).

Patients completed a paper headache diary 3 months before the anti‐CGRP mAb prescription and monthly throughout the treatment period, recording MMDs and the use of acute medications, the HIT‐6 score monthly, and the MIDAS score every 3 months. Due to the exploratory design and post hoc analysis, no formal sample size calculation was performed.

The last month of treatment was defined as (i) the completion of 12 months of follow‐up or (ii) the last month prior to discontinuation (i.e., the last administration). Data distribution was evaluated with the Shapiro–Wilk test. We reported the mean for continuous variables and the number (percentage) for categorical data. No imputation was done for missing data. A chi‐squared test for association and a point‐biserial analysis for correlation were conducted.

For the point‐biserial analysis, there were (a) no outliers, as assessed by boxplot; (b) engagement score was normally distributed, as assessed by Shapiro–Wilk's test (*p* > 0.05); and (c) there was homogeneity of variances, as assessed by Levene's test for equality of variances. In the chi‐squared test for association, all expected cell frequencies were greater than five.

PGIC score was reported as an ordinal variable (1 [no improvement] to 7 [maximum improvement] score). Furthermore, patients were considered responders if the PGIC score was 5 or greater (categorized PGIC score) at month 12, according to Pozo‐Rosich et al. [10]. The proportion of responders was measured in the reduction from baseline in MMDs as responders (≥ 50% response rate cutoff) or categorized as (0%–29%, 30%–49%, 50%–75%, 75%–99%, and 100% reduction in MMDs).

## 3 Results

We included 169 patients (137 females, 81.1%) treated with anti‐CGRP mAbs with a potential 12 months follow‐up regardless of discontinuation. The cohort comprised 133 chronic patients (78.7%) with mean MHDs of 20.9 ± 7.8 (demographical and clinical characteristics are summarized in Table 1).

**TABLE 1:** Patients demographic and clinical features at baseline.

	Overall population (*n* = 169)
Demographics	
Age (years), mean ± SD	46.6 ± 14.2
Sex female, *n* (%)	137 (81.1)
Type of anti‐CGRP mAb	
Erenumab, *n* (%)	56 (33.1)
Galcanezumab, *n* (%)	69 (40.8)
Fremanezumab, *n* (%)	44 (26.0)
Migraine features	
Age at onset, mean ± SD	16.6 ± 9.1
Aura, *n* (%)	15 (8.9)
Chronic migraine, *n* (%)	133 (78.7)
Medication overuse, *n* (%)	126 (74.6)
Monthly headache days, mean ± SD	20.9 ± 7.8
Absolute number of analgesics, mean ± SD	24.1 ± 20.3
Days with use of analgesics, mean ± SD	16.0 ± 8.4

*Note:* Percentages are expressed on column total.

Abbreviations: CGRP, calcitonin gene‐related peptide; mAb, monoclonal antibody; SD, standard deviation.

Thirty‐six patients (36/169, 21.3%) discontinued treatment for ineffectiveness. In the last month of treatment (regardless of early discontinuation or completion of 12 months of treatment), PGIC responders (5 or greater score) were 118/169 (69.8%), and MMDs responders (≥ 50% reduction in MMDs) were 100/169 (59.2%).

A chi‐squared test for association was conducted between discontinuation and PGIC responders or MMD responders. Both associations were statistically significant, MMD responders *χ* ^2^ = 29.884, *p* < 0.0001, as well as PGIC responders *χ* ^2^ = 33.474, *p* < 0.0001. There was a moderately strong association between both variables and discontinuation, with PGIC responders slightly higher (*φ* = 0.445, *p* < 0.0001) compared to MMD responders (*φ* = 0.421, *p* < 0.0001).

Therefore, we investigated the association between the overall PGIC (1 to 7 scale) and response rates. Point‐biserial correlations were run between discontinuation and PGIC score or categorized response rates. There was a stronger statistically significant correlation between PGIC score and discontinuation (*r*_pb_ = 0.541, *p* < 0.0001) compared to response rates and discontinuation (*r*_pb_ = 0.470, *p* < 0.0001), with patients that discontinued showing a lower PGIC score (3.33 ± 2.13 vs. 5.72 ± 1.32) and a higher percentage of patients in the 0–30 response rate category (66.7% vs. 15.9%).

## 4 Discussion

In our study, treatment discontinuation was significantly linked to both MMD response rates and PGIC scores, with PGIC demonstrating a strong correlation. These findings suggest the importance of PROMs in defining treatment response to anti‐CGRP mAbs.

Pain is a subjective experience that is difficult to assess thoroughly. It is well known that some patients may be satisfied by preventive treatments just by experiencing a reduction in pain intensity and associated symptoms [9].

In our recent study, several parameters (such as MHDs, analgesics use, MIDAS, and HIT‐6) showed statistically significant reductions during treatment even in patients who discontinued anti‐CGRP mAbs at some point for “ineffectiveness,” suggesting at least some effect of the treatment, though not sufficient to distinguish patients that continue vs. patients that discontinue treatment clearly [11]. In a study assessing the difference between MIDAS and MMDs after 3 months of treatment with erenumab, MIDAS responders only partly reflect the 12‐month outcome treatment in chronic migraine patients, as it excludes more than one‐third of responders [12].

Therefore, while some variables are classified as more or less useful for defining a clinical response, a single parameter can hardly capture the multifaceted action of anti‐CGRP mAbs and the complex symptomatology of migraine beyond pain [9].

For this reason, the use of composite scores or algorithms that consider multiple variables to evaluate the response to preventive treatments, particularly anti‐CGRP therapies, is increasingly emerging in migraine research [13].

Over the years, objective and quantitative endpoints have been complemented by PROMs, defined as self‐reported measures of symptoms, functional status, and perceptions [9]. PROMs are important tools for capturing information from the patient's perspective and should be used to evaluate the impact on quality of life, functional and emotional disability, and, to track patients' progress after starting a preventive treatment [9].

A recent study that compared different PROMs to evaluate treatment improvement demonstrated that the migraine‐specific quality of life questionnaire (MSQ) was the PROM that was significantly correlated with an improvement in all the treatment efficacy outcomes after 12 weeks of anti‐CGRP‐mAbs. In addition, a change in the MSQ and PGIC scores was the most accurate way of predicting the continuation of CGRP‐mAbs treatment at week 12 [7].

Therefore, a good option to evaluate treatment response, especially for anti‐CGRP treatments, could be to use PROM scales that better reflect improvement across treatment outcome measures, such as MSQ and PGIC, as also supported by this study.

At the same time, PROMs are inherently limited and subject to considerable variability due to factors such as their brevity, interpretive challenges, and the respondent's educational background [14].

In conclusion, the use of standardized and validated questionnaires is essential in clinical studies, particularly those involving migraine patients. It is crucial to account for multiple variables when evaluating the effectiveness of preventive migraine treatments, especially in light of the availability of CGRP‐targeted therapies. Such advancements could enhance the optimization of novel anti‐CGRP treatments, minimize inappropriate discontinuation, and ultimately improve outcomes for all patients with migraine.

## Author Contributions

All authors critically reviewed the manuscript, agreed to be fully accountable for ensuring the integrity and accuracy of the work, and read and approved the final manuscript.

## Ethics Statement

The study was approved as a part of the *Registro Italiano Cefalee* (RICe) study by the local Ethics committee (CEAVC Studio RICe, 14591_oss and subsequent amendments 2022‐609; 2023).

## Conflicts of Interest

L.F.I. received fees and Honoraria for advisory boards and speaker panels from Teva, Eli Lilly, Lundbeck, Pfizer, and AbbVie. C.V. received fees, Honoraria for advisory boards, support for attending meetings and/or travel, and speaker panels from Teva, Eli Lilly, Lundbeck, Pfizer, and AbbVie. M.R. received support for attending meetings and/or travel from AbbVie and Lundbeck. P.C. received fees, Honoraria for advisory boards, support for attending meetings and/or travel, and speaker panels from AbbVie, Bayer Schering, Bial, Biogen‐Dompè, Biogen‐Idec, Eisai, Genzyme, Lundbeck, Lusofarmaco, Merck‐Serono, Novartis, Prexton, Teva, UCB Pharma, and Zambon.

## Data Availability

The data collected and analyzed for the current study are available from the corresponding author upon reasonable request.
